# Supplementary material for: Betavulgarin Isolated from Sugar Beet (Beta vulgaris) Suppresses Breast Cancer Stem Cells through Stat3 Signaling
Source: Molecules. 2020 Jun 30;25(13):2999. doi: 10.3390/molecules25132999 (PMC7412145; doi:10.3390/molecules25132999)
Supplement: Supplementary file 1 [file molecules-25-02999-s001.pdf]

Table S1. Specific Real-time RT-qPCR primer sequences containing *Nanog, Sox2, Oct4, C-myc and β-actin* genes

Genes	Primers
Nanog	Forward: 5′-ATGCCTCACACGGAGACTGT-3′<br>Reverse: 5′-AAGTGGGTTGTTTGCCTTTG-3′
Sox2	Forward: 5′-TTGCTGCCTCTTTAAGACTAGGA-3′<br>Reverse: 5′-CTGGGGCTCAAACTTCTCTC-3′
Oct4	Forward: 5′-AGCAAAACCCGGAGGAGT-3′<br>Reverse: 5′-CCACATCGGCCTGTGTATATC-3′
C-myc	Forward : 5'-CCTGGTGCTCCATGAGGAGAC-3'<br>Reverse : 5'-CAGACTCTGACCTTTTGCCAGG-3'
β-actin	Forward: 5’-TGTTACCAACTGGGACGACA-3’<br>Reverse: 5’-GGGGTGTTGAAGGTCTCAAA-3

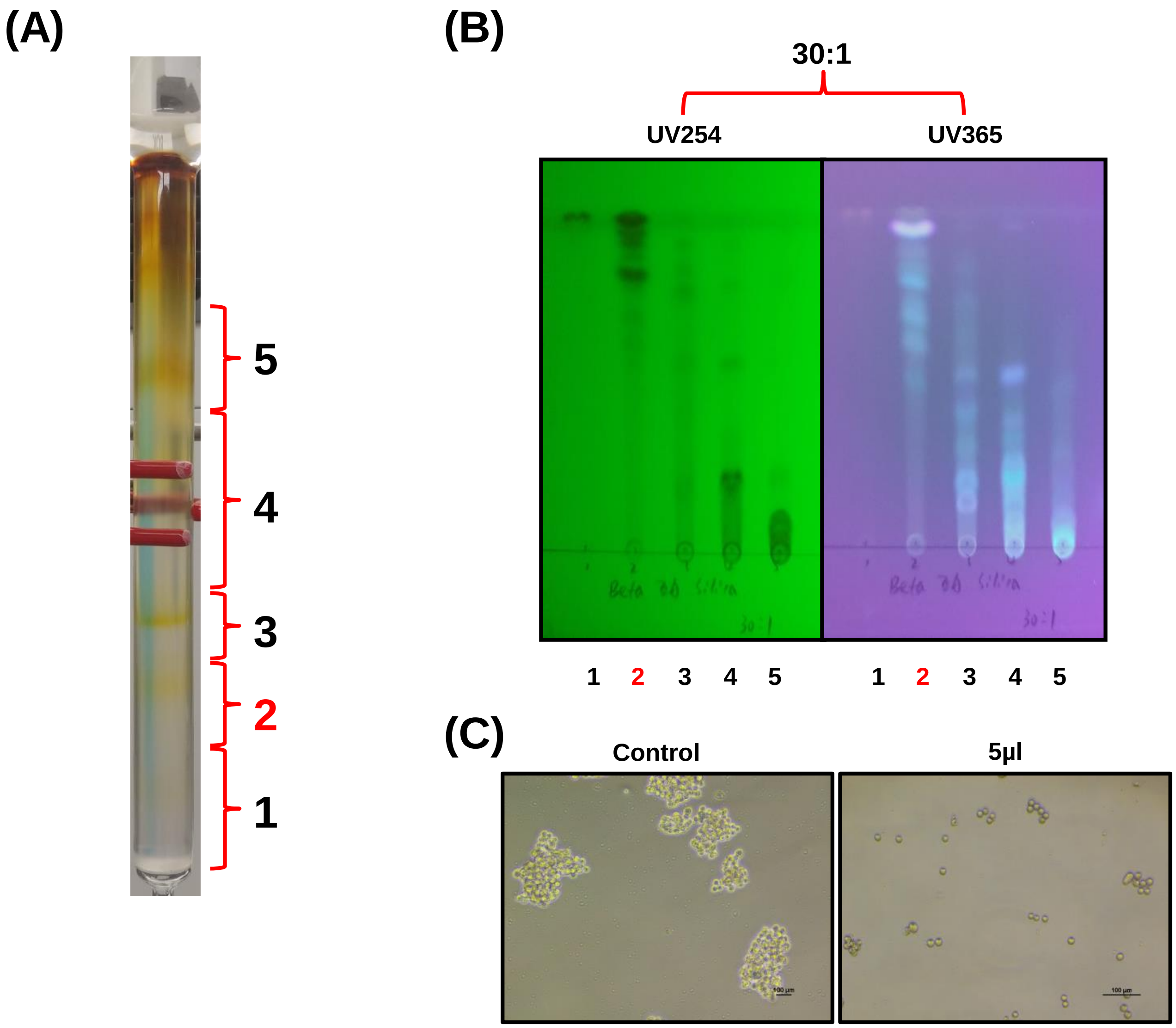

Figure S1. The purification procedure of a mammosphere formation inhibitor derived from beet using silica gel column chromatography. (A) The sample was isolated by silica gel chromatography with a solvent mixture [$CHCl_3$: MeOH (10:1)]. (B) TLC plate analysis of the purified sample ($CHCl_3$: MeOH = 30:1). Active fraction: #2. (C) Mammosphere formation assay using the purified fraction #2.

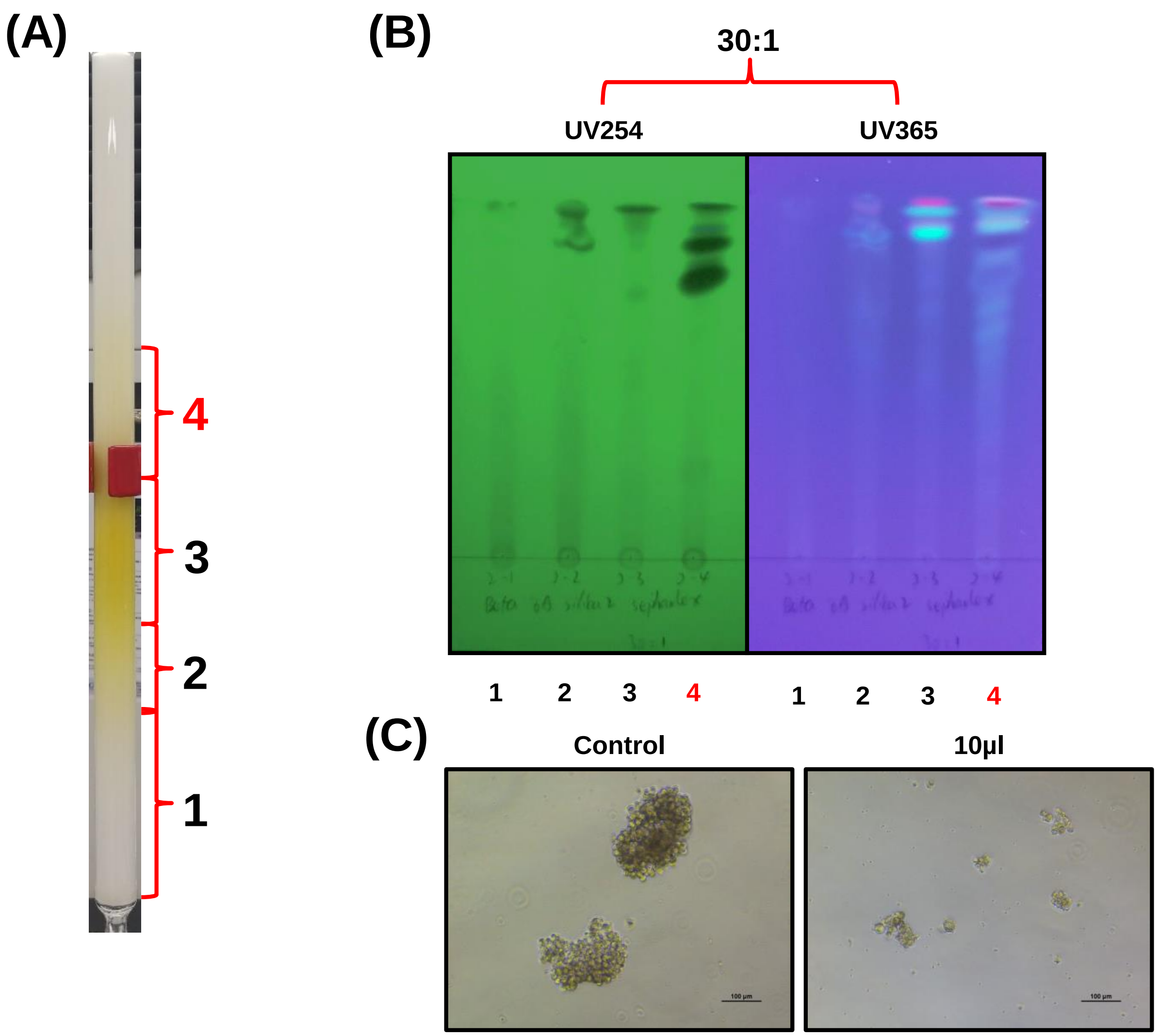

Figure S2. The purification procedure of a mammosphere formation inhibitor derived from beet using Sephadex LH-20 column chromatography. **(A) The sample was isolated by Sephadex LH-20 chromatography with MeOH. (B) TLC plate analysis of the purified sample ($CHCl_3$: MeOH = 30:1). Active fraction: #4. (C) Mammosphere formation assay using the purified fraction #4.**

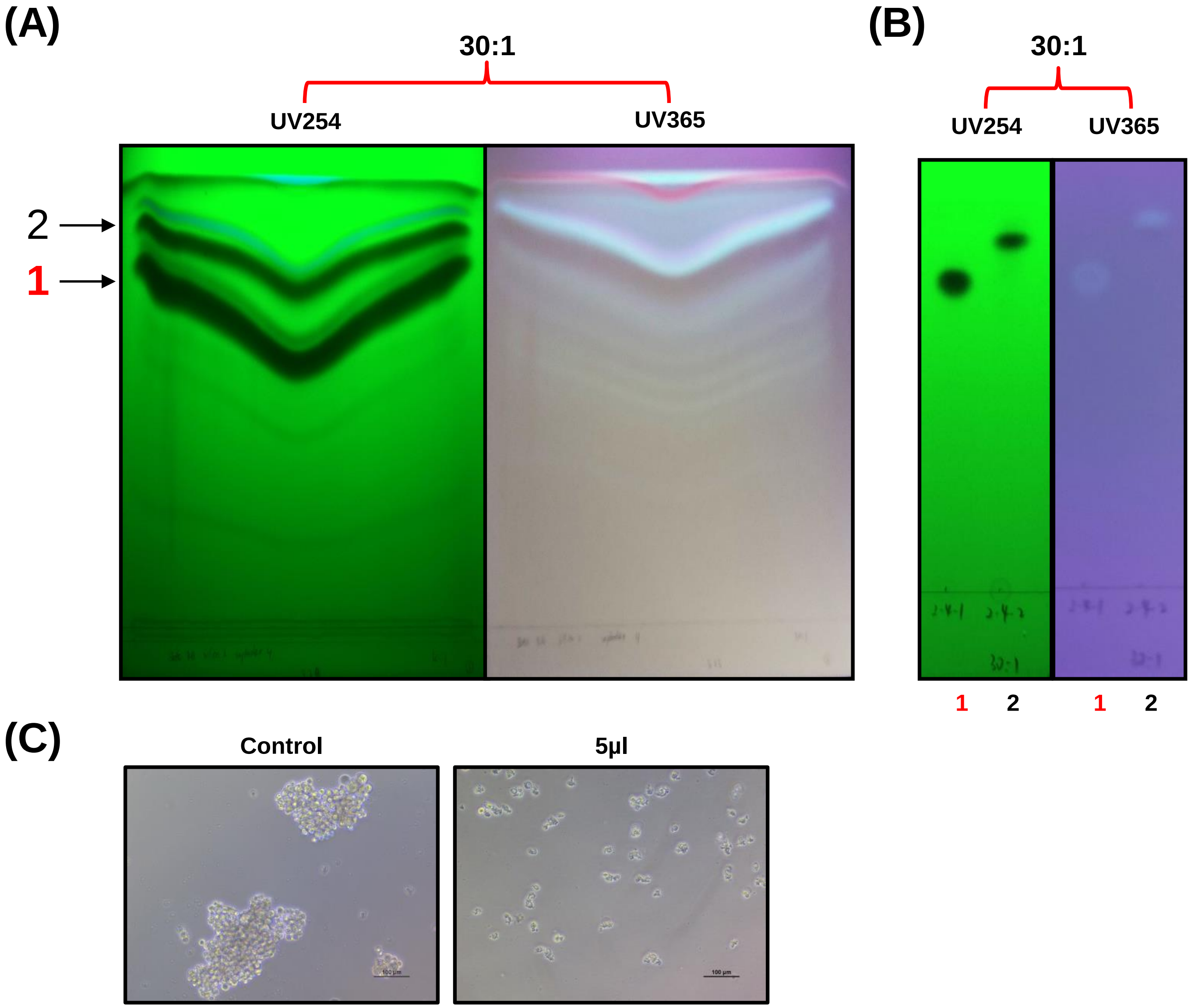

Figure S3. The purification procedure of a mammosphere formation inhibitor derived from beet using preparative thin layer chromatography with $CHCl_3$:MeOH (30:1). (A) Preparatory TLC chromatography containing fractions 1 and 2. (B) TLC plate analysis of the prepared TLC bands after the samples were scraped and purified (fractions 1 and 2, $CHCl_3$: MeOH = 30:1). Active fraction: #1. (C) Mammosphere formation assay using the purified fraction #1.

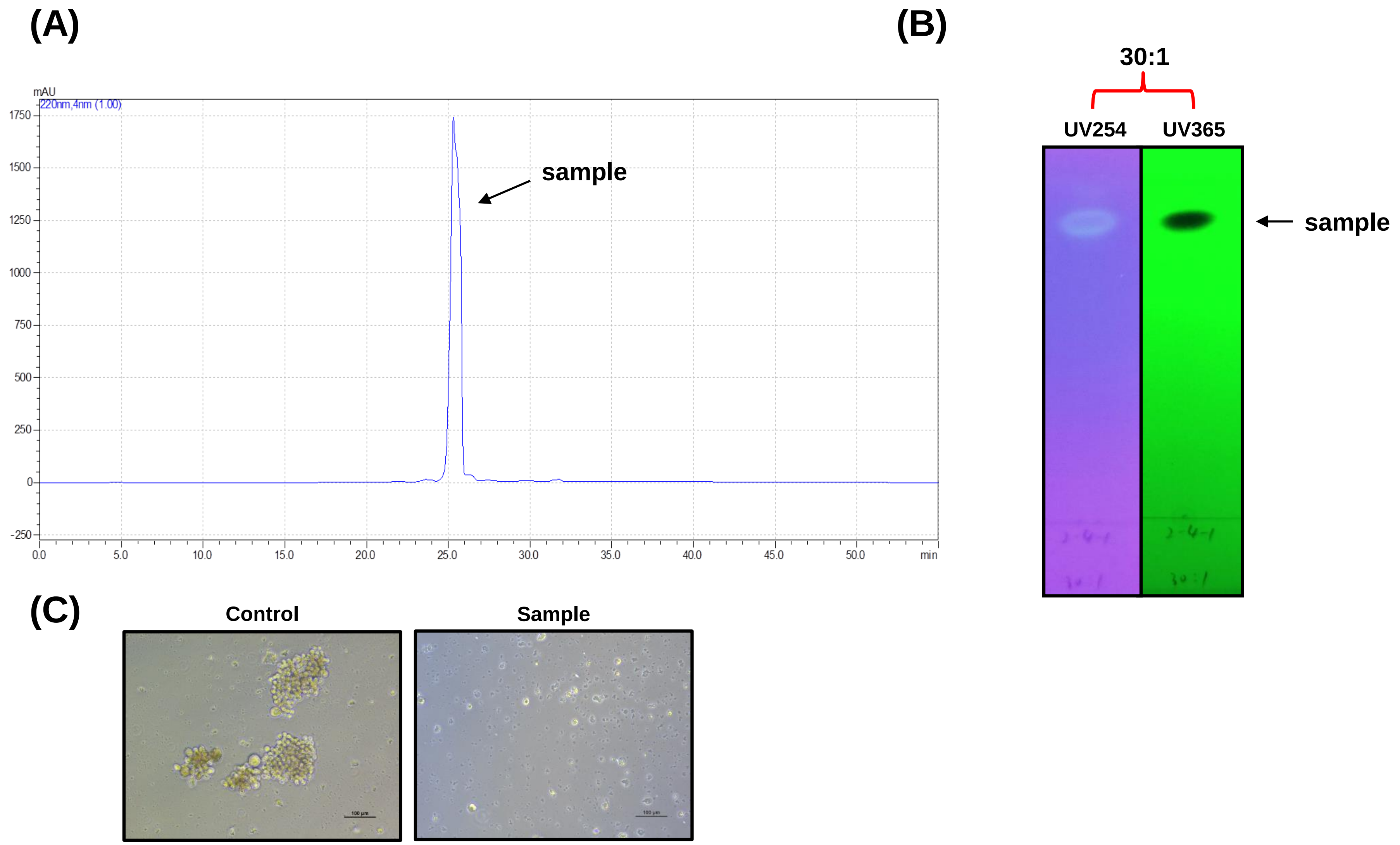

Figure S4. Purification procedure of a mammosphere formation inhibitor derived from beet using HPLC. (A) Assessment of the major fractions using HPLC at two wavelengths. Samples were collected based on the 254 and 220 nm wavelengths. (B) TLC plate analysis of the purified sample ($CHCl_3$: MeOH = 30:1). (C) Mammosphere formation assay using the purified sample.

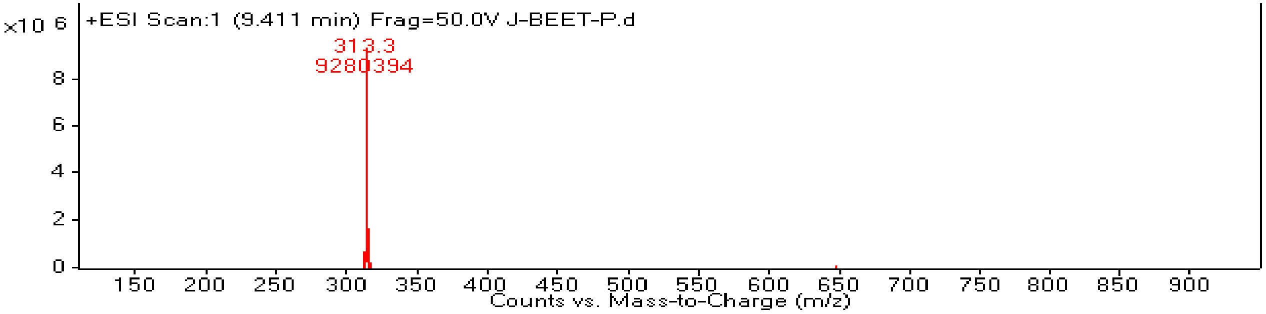

Figure S5. ESI-mass spectrum in positive mode of the purified sample

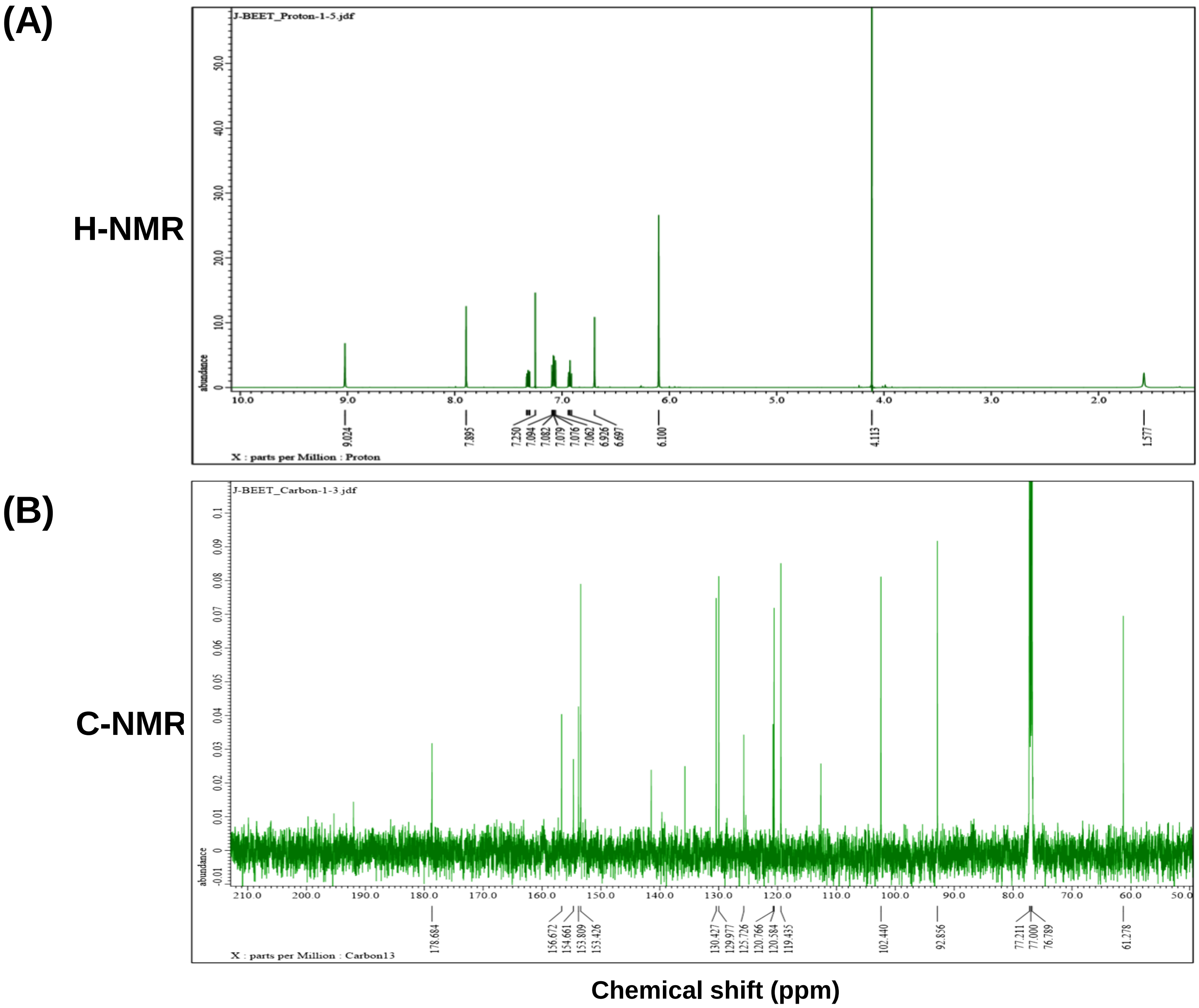

Figure S6. ^{1}H **NMR and** ^{13}C **NMR spectra of purified sample**

HMQC

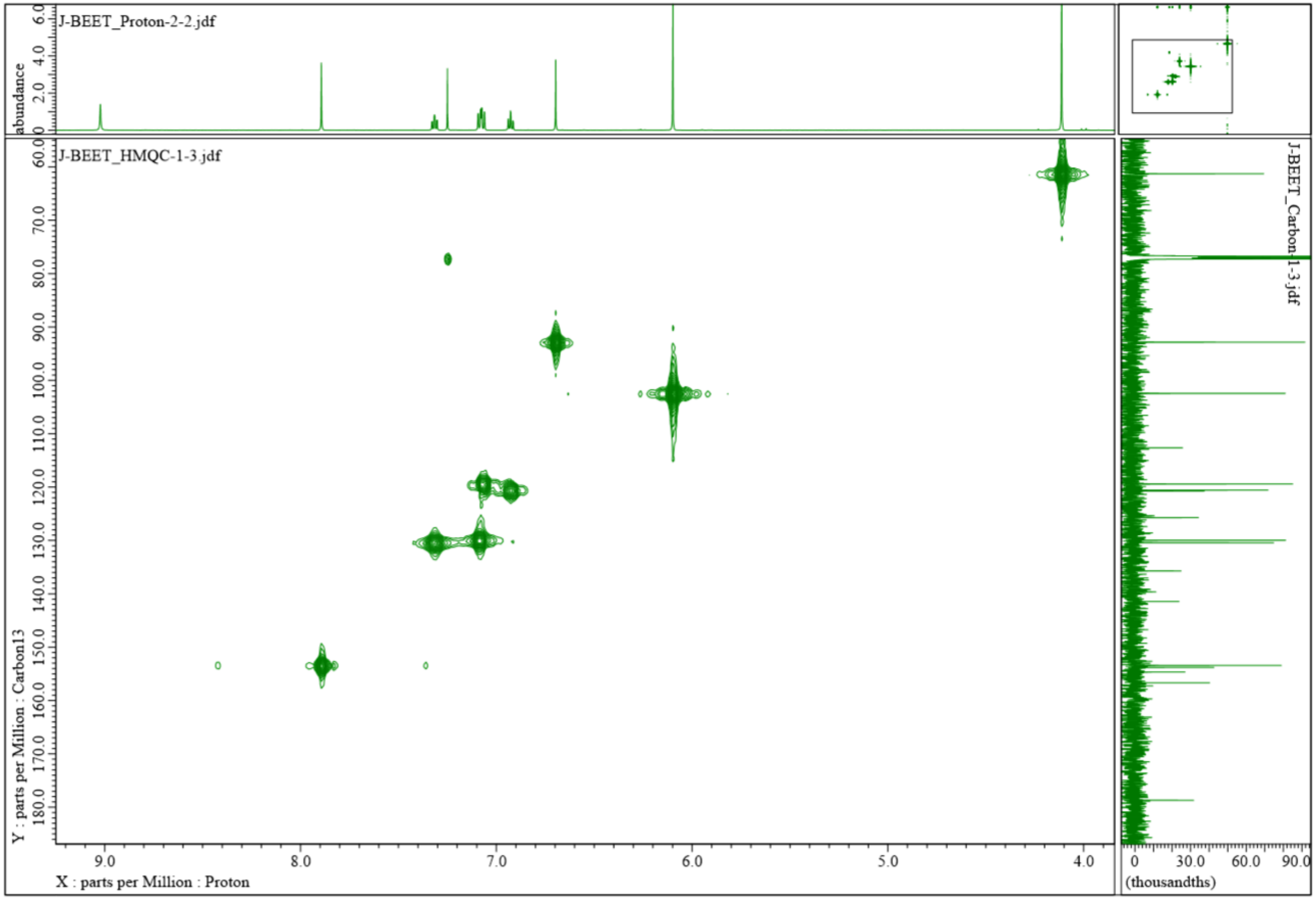

Figure S7. HMQC spectrum of the purified sample.

COSY

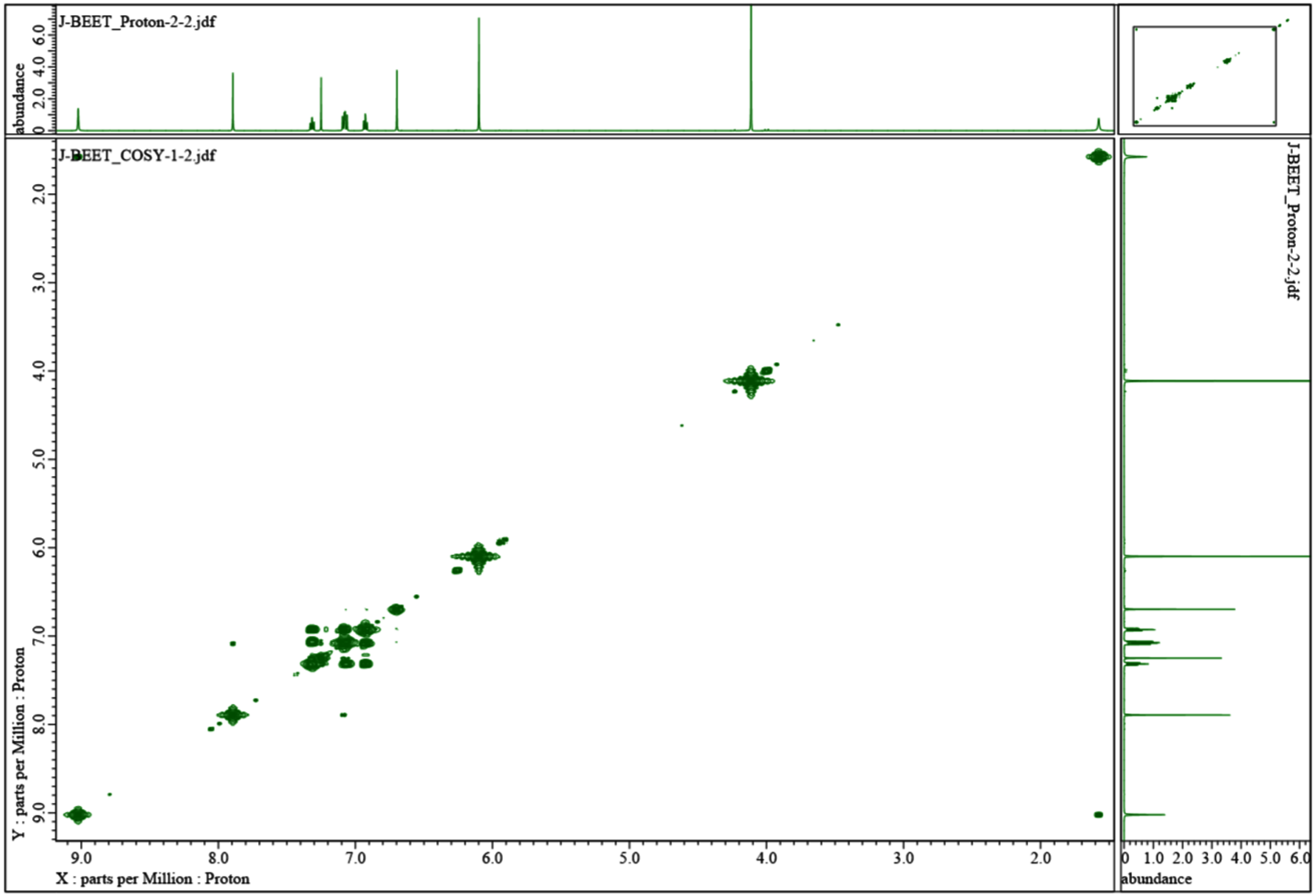

Figure S8. ^{1}H-^{1}H COSY spectrum of the purified sample.

HMBC

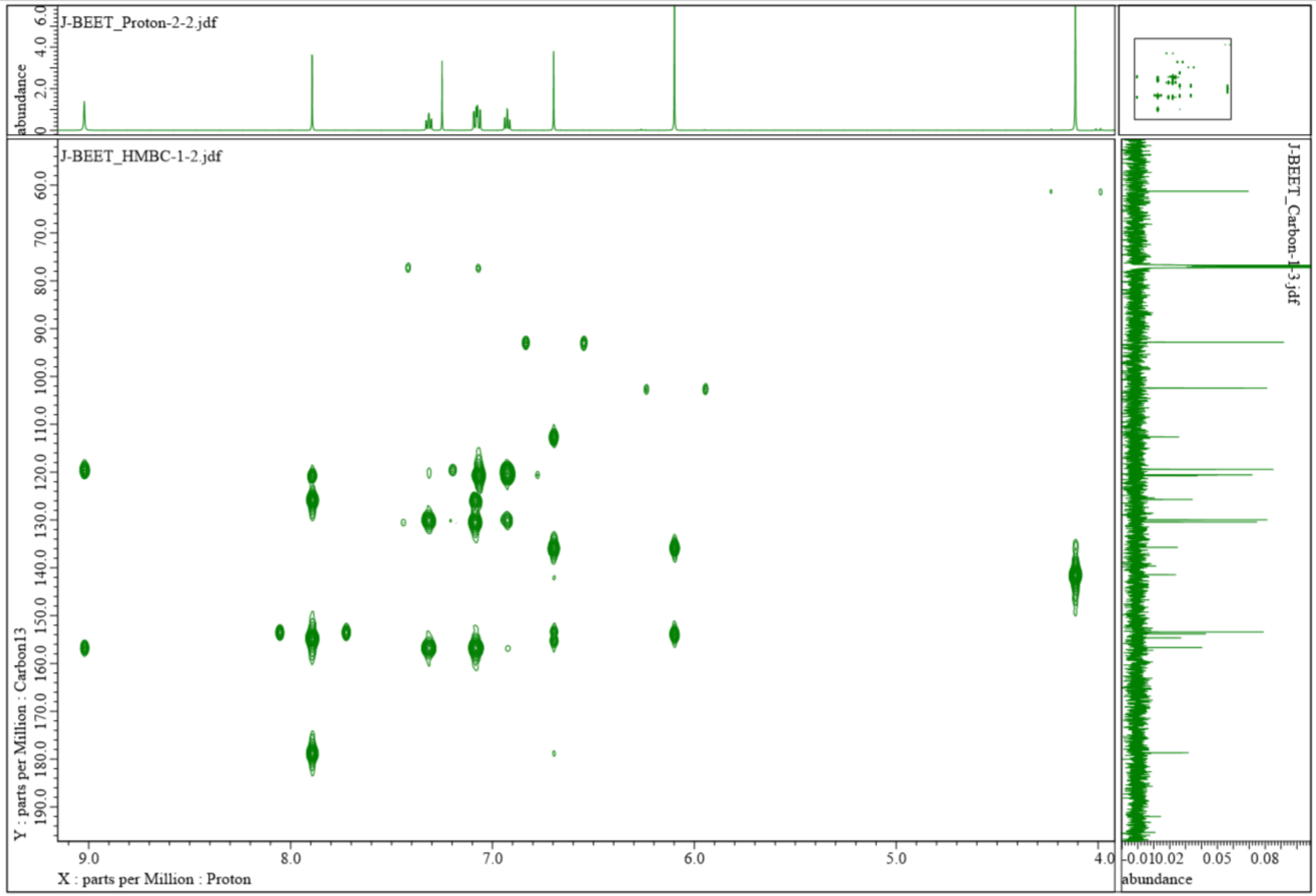

Figure S9. HMBC spectrum of the purified sample.

(A)

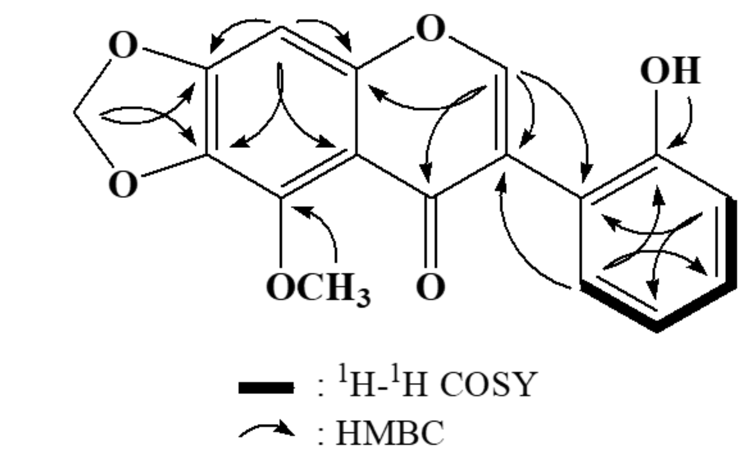

(B)

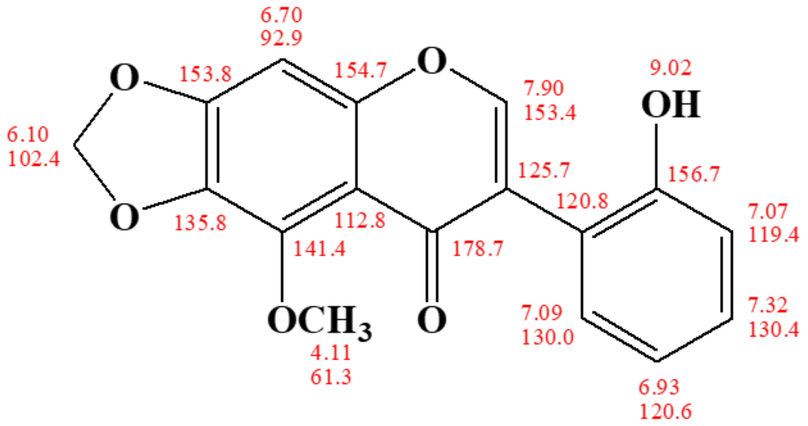

Betavulgarin: $C_{17}H_{12}O_6$, Molecular weight: 312

Figure S10. Two-dimensional NMR data (A) and ^{1}H and ^{13}C peaks assignments (B) of the purified sample
